# Effect of ionic strength and presence of serum on lipoplexes structure monitorized by FRET

**DOI:** 10.1186/1472-6750-8-20

**Published:** 2008-02-26

**Authors:** Catarina Madeira, Luís MS Loura, Manuel Prieto, Aleksander Fedorov, M Raquel Aires-Barros

**Affiliations:** 1 IBB-Institute for Biotechnology and Bioengineering, Centre for Biological and Chemical Engineering, Av. Rovisco Pais, 1049-001, Portugal; 2 Faculdade de Farmácia, Universidade de Coimbra, Rua do Norte, 3000-295 Coimbra, Portugal; 3 Centro de Química de Évora, Rua Romão Ramalho, 7000-671 Évora, Portugal; 4 Centro de Química-Física Molecular, Complexo I, Instituto Superior Técnico, Av. Rovisco Pais, 1049-001, Portugal

## Abstract

**Background:**

Serum and high ionic strength solutions constitute important barriers to cationic lipid-mediated intravenous gene transfer. Preparation or incubation of lipoplexes in these media results in alteration of their biophysical properties, generally leading to a decrease in transfection efficiency. Accurate quantification of these changes is of paramount importance for the success of lipoplex-mediated gene transfer *in vivo*.

**Results:**

In this work, a novel time-resolved fluorescence resonance energy transfer (FRET) methodology was used to monitor lipoplex structural changes in the presence of phosphate-buffered saline solution (PBS) and fetal bovine serum. 1,2-dioleoyl-3-trimethylammonium-propane (DOTAP)/pDNA lipoplexes, prepared in high and low ionic strength solutions, are compared in terms of complexation efficiency. Lipoplexes prepared in PBS show lower complexation efficiencies when compared to lipoplexes prepared in low ionic strength buffer followed by addition of PBS. Moreover, when serum is added to the referred formulation no significant effect on the complexation efficiency was observed. In physiological saline solutions and serum, a multilamellar arrangement of the lipoplexes is maintained, with reduced spacing distances between the FRET probes, relative to those in low ionic strength medium.

**Conclusion:**

The time-resolved FRET methodology described in this work allowed us to monitor stability and characterize quantitatively the structural changes (variations in interchromophore spacing distances and complexation efficiencies) undergone by DOTAP/DNA complexes in high ionic strength solutions and in presence of serum, as well as to determine the minimum amount of potentially cytotoxic cationic lipid necessary for complete coverage of DNA. This constitutes essential information regarding thoughtful design of future *in vivo *applications.

## Background

The conditions in which DNA-cationic lipid complexes (lipoplexes) are prepared may affect greatly their final structure. In transfection studies, lipoplexes contact physiological saline concentrations and serum, which is known as the first barrier to cellular delivery [1]. A liposomal formulation with high condensation efficiency of DNA in low ionic strength solutions may not display the same behaviour when placed in physiological saline solutions and serum. On the other hand, the study of the interactions of lipid vectors with serum may serve as a predictive model for their *in vivo *efficiencies.

The destabilizing effect of physiological saline solutions in the lipoplex structure, either when they are prepared in these solutions or added to them after being prepared in low ionic strength solutions, has been reported [2,3]. It has been verified that although high salt concentration weakens the association between lipid and DNA, with the occurrence of some dissociation, the complexes remain largely intact [4].

Serum has been proposed as an important barrier to cationic-lipid mediated intravenous gene transfer [5]. The interaction of lipoplexes with serum results in alteration of their biophysical properties, leading to a decrease in transfection efficiency, and several studies have been carried out in order to understand the magnitude of these changes. Disruption of the complex does not occur when it is in contact with plasma and therefore this is not the reason for the loss of transfection activity [6]. Instead, coating of complexes with plasma components seems to cause reduced uptake by cells, which in turn results in reduced transfection. The lipid-associated proteins present in serum seem to be mostly responsible for the low transfection efficiencies [7].

Due to their versatility and sensitivity, fluorescence techniques have been widely used in the characterization of lipoplex structure. Among them, Fluorescence (or Förster) Resonance Energy Transfer (FRET) is particularly suited to this purpose, because of its critical dependence on proximity between donor and acceptor fluorophores and its ability to measure distances in the nanometer scale [8]. Recently, the first application of a FRET method to evaluate the condensation state of plasmid DNA in lipoplexes in the presence of serum (using two DNA probes) was reported [9]. In order to evaluate lipoplexes' stability in serum, a FRET study using a lipid probe as donor and a DNA-located acceptor was subsequently published [10]. In these studies, lipid-DNA interactions were qualitatively monitored, and no attempt to analyze the FRET data with a photophysical model was made.

In the present study, time-resolved FRET is used in a quantitative way to evaluate lipoplex stability in serum and to our knowledge it is the first time that this is reported. To this purpose, the theoretical formalism describing the changes in donor (DNA-bound probe) fluorescence in presence of nearby acceptors (membrane probe molecules), assuming a multilamellar arrangement (which is maintained even in the presence of serum [11]) is applied to the fitting of the variation of FRET efficiency as a function of acceptor concentration. For each lipoplex formulation under study (that is, for each charge ratio and buffer medium under study), the donor-acceptor interplanar distances and the amount of free DNA are recovered.

## Results and discussion

In transfection studies, lipoplexes contact with physiological saline conditions and serum. While some lipoplex preparation methods include hydration of the lipid film and DNA in physiological saline buffers [12], others use water or low ionic strength saline solutions [13]. In our study, in order to compare the degree of lipoplex dissociation in these two situations ((*i*) lipoplexes prepared in PBS; and (*ii*) lipoplexes prepared in Tris-HCl followed by incubation in PBS), the extent of coverage of a 6717 bp plasmid by DOTAP (complexation efficiency) was measured for several charge ratios. Since in *in vitro *transfection studies lipoplexes are usually incubated in serum, evaluation of lipoplex behaviour in a 10% serum solution was also carried out.

### Steady-state and time-resolved fluorescence results

**Figure 1:**
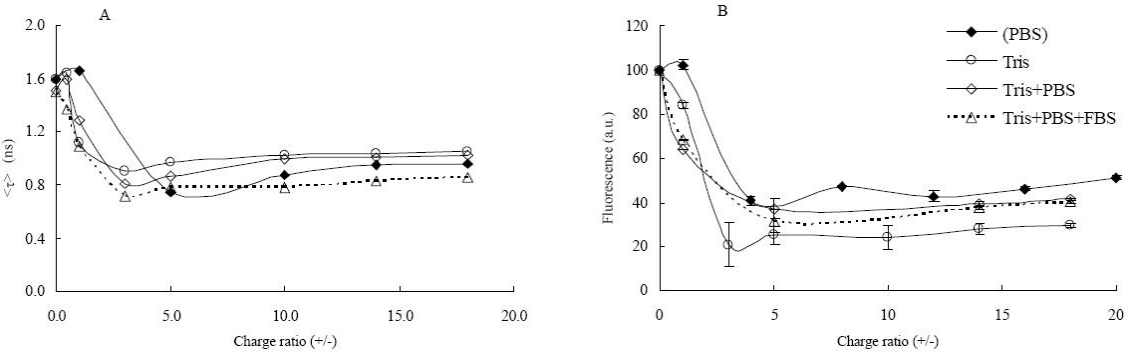
**Fluorescence data of BOBO-1 intercalated on DNA within DOTAP liposomes**. Fluorescence lifetime weighted quantum yield (A) and intensity (B). Lipoplexes prepared in Tris-HCl, pH 7.4 (-○-); Lipoplexes prepared in PBS (-◆-); Lipoplexes prepared in Tris-HCl and incubated on PBS (-△-); Lipoplexes prepared in Tris-HCl, incubated on PBS, followed by incubation on FBS (-□-).

Fluorescence decays of BOBO-1 within lipoplexes were measured to get further information on the changes in probe microenvironment induced by complexation with DNA, and the lifetime-weighted quantum yield values (see Methods section) obtained for each charge ratio are shown in Fig. 1A. Three exponentials are needed to describe BOBO-1 decays, and the presence of liposomes leads to a drastic decrease up to (+/-) ≈ 3. At higher charge ratios, a constant value of lifetime-weighted quantum yield is obtained in each of the studied situations.

The steady-state fluorescence intensity of BOBO-1 (Fig. 1B) shows a similar variation upon DNA complexation, and in all cases a ~2 to 5-fold decrease was observed at higher charge ratios, indicating that BOBO-1 fluorescence is greatly affected by the presence of cationic liposomes. For these charge ratios, the difference between the fluorescence intensities of BOBO-1 in lipoplexes prepared in PBS and in Tris-HCl may be due to a higher amount of BOBO-1 still intercalated in DNA in the former system.

### Lipoplex complexation efficiencies and interchromophore spacing distances

**Figure 2:**
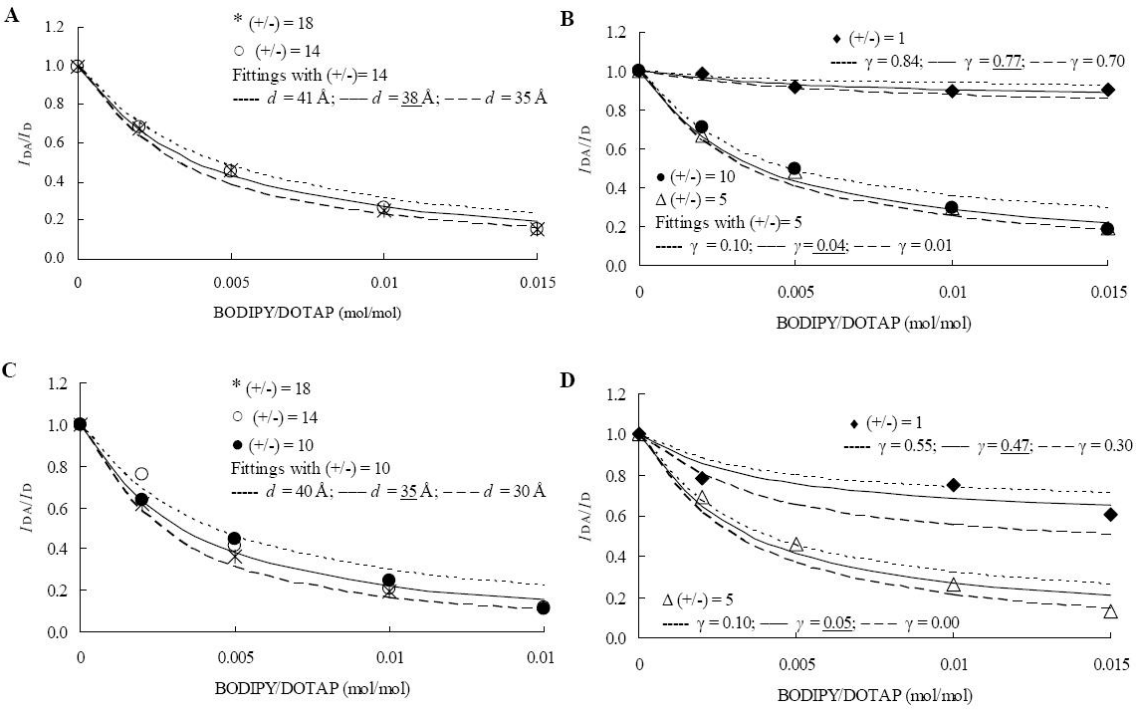
**FRET curves obtained with different ionic strength conditions**. FRET quenching ratios, *I*_DA_/*I*_D _= 1-*E*, for BOBO-1/BODIPY-PC pairs in DOTAP/DNA prepared in PBS (A,B) and in Tris-HCl, followed by incubation in PBS (C,D). Fitting curves using Eqs. 2–3 are also shown.

In our experimental design, FRET between the DNA-bound donor and the membrane-located acceptor only occurs if the two fluorophores are sufficiently near each other (less than two times the Förster distance, *R*_0_), and therefore an increase in FRET efficiency with acceptor concentration is a direct evidence of coverage of DNA by the labeled liposomes. This is clearly observed for all studied systems, as shown in figs. 2A–D. The experimental results were compared with the theoretical curves obtained with Eqs. 2–3, and either the donor-acceptor interplanar distance *d *or the fraction of free DNA *γ *were recovered, as explained in detail in the Methods section (FRET assays). The complexation efficiencies, shown in Table 1, were calculated using the underlined *d *and *γ *values shown in Fig.2.

**Table 1:** FRET model fitting parameters (interchromophore spacing distance and complexation efficiency) for the studied systems.

	Donor-acceptor distance			Complexation efficiency (C.E) = (1 - *γ*) × 100	
			
Lipoplex Preparation/incubation	(+/-)	*d *(Å)	*R*_0 _(Å)	(+/-)	C.E (%)
PBS	14–18	38	43.0	14	100
				10	97
				5	96
				1	23

Tris-HCl	5–18	40	42.0	5	100
				1	60
				0.5	25

Tris-HCl/PBS	10–18	35	43.0	10	100
				5	95
				3	90
				1	53
				0.5	23

Tris-HCl/PBS+FBS	14–18	30	42.0	14	100
				10	94
				5	94
				3	89
				1	55
				0.5	15

When lipoplexes are prepared in PBS rather than in low ionic strength buffer, 23% of DNA is covered by the liposomes at charge ratio (+/-) = 1, whereas lipoplexes prepared in Tris-HCl followed by addition of PBS have 53% of protected DNA for the same charge ratio (Fig 2D). Furthermore, lipoplexes prepared in Tris-HCl have an even higher degree of complexation of DNA (60%). On the other hand, and according to our results, complexation was virtually complete for (+/-) = 5 in all the studied conditions, complexation efficiencies ranging from 95% to 100% being obtained (see Table 1 and Fig 2B–D). A similar behaviour was observed with other dyes, in a qualitative FRET assay, using NaCl concentrations ranging from 100–500 mM [4].

The quantification of DNA displacement upon contact with physiological saline solutions is often described in the literature as being made using destructive methods such as fluorescence measurements after lipoplex ultracentrifugation using sucrose gradients [13-15] or agarose gel electrophoresis [10,16,17]. Our results show a clear displacement of DNA from lipoplexes upon contact with physiological ionic strength media, and, to our knowledge is the first time that this quantification is made using a non-destructive methodology. In most cases, comparison of these authors' results with our own is not straightforward, because of differences in the lipid composition and/or buffer medium of the systems under study. One work for which direct comparison is possible is that of Szoka *et al*. [13], who studied (among other formulations) lipoplexes prepared in low ionic strength (30 mM Tris-HCl) with DOTAP as the sole lipid component. These authors observed that above (+/-) = 5 the lipid/nucleotide ratios in the separated lipoplexes were invariant, denoting complete DNA condensation, is in full accordance with our results (see Table 1, second line).

Given that the theoretical curves of Fig. 2 describe our results adequately, we expect that a lamellar lipoplex structure is formed in all studied systems, though with slightly different interlamellar distances. With lipoplexes prepared in PBS, a 38 Å intercromophore distance (*d*) was recovered from FRET curves at very high charge ratios (+/-) = 14 and 18 (see Fig. 2A). This lower value, when compared with 40 Å obtained with lipoplexes prepared in Tris-HCl, probably arises solely from the decrease of the DNA superhelix amplitude in the presence of physiological saline solutions, previously confirmed by others [18]. In contrast, when PBS is added to pre-formed lipoplexes (in 30 mM Tris-HCl) an even smaller interchromophore spacing distance is recovered (35 Å; see Fig. 2C). This distance is common to several charge ratios of lipoplexes prepared in the same manner ((+/-) = 10, 14 and 18). The osmotic shock sensed by the lipoplexes when they are placed in a higher ionic strength solution may be responsible for this decrease. The exit of solvent within the lipoplex when in contact with higher ionic strength solutions, contributing to a small dehydration at the lipid-water interface, was previously verified by other methods, such as differential scanning calorimetry, filtration and dialysis [19]. In an apparently divergent statement, Wiethoff et al. verified an increase on the donor-acceptor dye distance of closest approach with the increase of ionic strength at charge ratios (+/-) above 3 [20]. Knowing that the presence of salt induces a smaller complexation efficiency for each given charge ratio, as quantified in our work, we can justify that apparent "distance increase" (inferred by a decrease on energy transfer efficiency) with the presence of free DNA, containing isolated donors.

### Effect of serum in the lipoplex stability

The instability of lipoplexes in serum, which is one of the possible causes of the obtained lower transfection efficiencies, is a well-known phenomenon. Recently, several structural, physical-chemical and biophysical studies of lipoplexes in serum have been carried out to inquire about the causes of this instability [5,10,11,21,22]. By understanding this problem, it is easier to overcome it, and, with our work, we hope to contribute to the knowledge of lipoplex structural changes upon contact with serum. All types of serum more frequently used (FBS, BSA) contain several blood proteins including endonucleases. Whereas endonucleases degrade dsDNA, the function of specific protein association with lipoplexes is only beginning to be understood [21].

**Figure 3:**
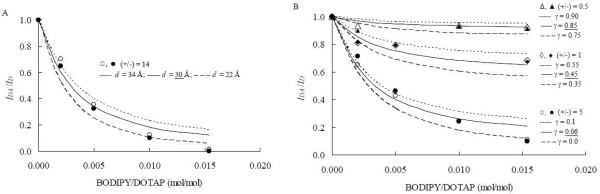
**FRET curves obtained after incubation in serum**. FRET quenching ratios, *I*_DA_/I_D _= 1-*E*, for BOBO-1/BODIPY-PC pairs in DOTAP/DNA prepared in Tris-HCl, incubated in PBS, followed by incubation in FBS for 30 min. (open symbols) and 2 h (filled symbols). Fitting curves using Eqs. 2–3 are also shown.

The presence of serum in lipoplexes previously incubated in PBS leads to the displacement of some DNA molecules, which is more pronounced for a charge ratio of 0.5, with complexation efficiency decreasing to 15% (against 23% verified in PBS and 25% in Tris-HCl, both in the absence of FBS, see Table 1). In lipoplexes with lower charge ratios, degradation of DNA by endonucleases upon contact with serum is probable. At higher charge ratios, similar complexation efficiencies were obtained when compared with the corresponding lipoplex formulation prepared in Tris-HCl and incubated in PBS. Upon serum addition, a multilamellar structure is still present, considering the good fit of the FRET data by the model, which assumes a multilamellar distribution of the dyes. This is verified for all charge ratios (Fig. 3). Identical values of interchromophore spacing distance (*d*) were recovered with lipoplex charge ratios (+/-) of 14 and 18, probably pointing to identical structures in both cases, with all DNA protected by the cationic liposomes. The invariance of BOBO-1 lifetime-weighted quantum yield at these charge ratios (see Fig. 1A) reflects the same environment sensed by the dye. However, these lifetime values are lower than those obtained in PBS or Tris-HCl and a smaller donor-acceptor spacing distance was recovered in presence of FBS (30 Å). It was previously shown that serum without certain lipoproteins is less inhibitory toward transfection efficiency of lipoplexes in several cell lines [7]. In agreement, it was also suggested that there is a possibility that lipids of lipoprotein particles interact and fuse with the cationic lipids of the lipoplex [21]. However, this protein-induced fusion will only occur at the lipoplex external layer, which would not drastically affect interchromophore spacing distances. The confirmation of protein binding to the lipoplexes external layer without either destabilizing the multilamellar structure [11], or dissociation [6,10,21] was previously reported. The multilamellar arrangement in serum and the respective recovered complexation efficiencies are maintained at least for 2 h, because the FRET experimental results for several charge ratios were almost identical after 30 min and 2 h incubation of lipoplexes in serum (Fig. 3).

## Conclusion

In this work a novel FRET methodology, based on computation of FRET efficiency from time-resolved fluorescence data and its analysis using a quantitative model consistent with the expected donor/acceptor distribution geometry (see Methods – FRET assays subsection below) is applied to the characterization of lipoplexes prepared under different conditions. The use of time-resolved instead of steady-state instrumentation eliminates common sources of error, such as inner filter artifacts [8]. On the other hand, and most importantly, correct quantitative model fitting of the FRET data enables the recovery of parameters of topological significance (*d *and *γ*), at variance with phenomenological applications of the technique.

The main result from this work is the application of our FRET methodology to monitor structural changes of DOTAP/pDNA lipoplexes, in physiological solutions, especially in serum, where interchromophore spacing distances and complexation efficiencies could be calculated. In some transfection protocols, lipoplexes are prepared in physiological saline solutions, whereas frequently they are prepared in low ionic strength solutions to avoid aggregation. However, in both cases, lipoplex contact with physiologic saline solutions is unavoidable, when the goal is a transfection procedure. In our study, both situations were investigated in terms of DOTAP complexation efficiency, and similar results were obtained. Complete DNA protection was verified at charge ratio 10 with lipoplexes prepared in Tris-HCl and incubated in PBS afterwards, whereas lipoplexes prepared in PBS with the same charge ratio have 97% of DNA covered by the liposomes. Differences of 3% complexation efficiency at this charge ratio are not significant, and an additional small extent of displacement of DNA (to a complexation efficiency of 94%) is observed when serum is present.

It is pertinent to address the relevance of our study, having in view *in vivo *applications. Looking at recent literature, and given the uncertainty surrounding the factors that affect lipofection, it is unsurprising that no particular charge ratio value is optimal for all systems. Although some authors found a charge ratio (+/-) of 2 as ideal for transfection with DOTAP [13], several others found higher charge ratios, in the 5–6 range, as the best for their respective systems [23,24]. Our results show that, in our studied conditions, charge ratios of ~5–10 ensure that all DNA is covered. However, as widely known, the use of an excess amount of lipid is detrimental in terms of lipid toxicity to the cells. Obviously, the transfection efficiency is determined by the combination of (among other factors) the extent of DNA protection afforded by the lipids and the degree of cytotoxicity due to the latter.

Taking this into account, it can be argued that our results agree with the widespread use of charge ratios in the 2–5 range in transfection. Looking at our complexation efficiencies (Table 1), it can be seen that whereas (+/-) = 1 does not ensure more than 60% of DNA protection, (+/-) = 5 ensures ≥ 94% of complexed DNA for all studied systems. Even (+/-) = 3 ensures ≥ 89% of protection for the systems where this charge ratio was studied (Tris-HCl/PBS and Tris-HCl/PBS+FBS). Therefore, the use of (+/-) in the 3–5 range will have little effect (~10% or less) regarding the extent of DNA protection compared to (+/-) = 10. However, these formulations will predictably be considerably milder regarding cytotoxicity. The combination of these two factors most probably results in these charge ratios being preferable to, say, (+/-) = 10 for the purpose of transfection. Our methodology can thus be used to predict which formulations will be most effective *in vivo*: those for which coverage of DNA is found to be sufficiently high (≥ 90%), without needing a large excess of cationic lipid.

Several literature studies [25,26] have shown that the use of lipid mixtures (e.g. including "helper lipids" such as phosphatidylcholine or phosphatidylethanolamine) could lead to improved lipoplex stability. DOTAP/DNA complexes form stable, multilamellar arrangements under a wide range of experimental settings [27]. We verified recently that inclusion of DOPE in the lipoplex formulation has little effect in lipoplex structure apart from a small reduction in the amount of cationic lipid required for complete DNA coverage, and, in particular, does not affect the recovered interchromophore spacing distances [28]. Therefore, we chose to study the effects of increased ionic strength and presence of serum using DOTAP as the sole lipid component at this stage. However, our future studies will use lipid mixtures in this context, and we plan to vary systematically the liposome composition in order to find the lipoplex formulations most resistant (in terms of showing higher complexation efficiencies for each charge ratio, and requiring the minimal amount of excess cationic lipid for extensive DNA coverage) to these perturbations.

## Methods

### Materials

The plasmid pVAX1lacZp25 with 6717 bp was constructed in the laboratory of the Centre of Biological and Chemical Engineering by M. Henriques, by cloning a gene that encodes for an envelope protein of the virus *Maedi-Visna *(p25) in the plasmid pVAX1LacZ (manuscript in preparation). The cationic lipid 1,2-dioleoyl-3-trimethylammonium-propane (DOTAP) was obtained from Avanti Polar Lipids (Alabaster, AL). The membrane probe 2-(4,4-difluoro-5-octyl-4-bora-3a,4a-diaza-s-indacene-3-pentanoyl)-1-hexadecanoyl-*sn*-glycero-3-phosphocoline (BODIPY-PC), as well as the DNA intercalating dye benzothiazolium, 2,2'-[1,3-propanediylbis[(dimethyliminio)-3,1-propanediyl-1(4H)-pyridinyl-4-ylidenemethy-lidyne]] bis [3-methyl]-tetraiodide (BOBO-1) were obtained from Molecular Probes (Eugene, OR). Liposomes and lipoplexes were prepared in 30 mM Tris(hydroxymethyl)-aminomethan buffer, pH 7.4 adjusted with hydrochloric acid (Tris-HCl) or in PBS (Phosphate-buffered saline: 150 mM NaCl, 16 mM Na_2_HPO_4_, 4 mM NaH_2_PO_4_, pH 7.4). All salts were of analytical grade and were obtained from Merck (Darmstadt, Germany). Fetal Bovine Serum (FBS) was kindly given by Prof. Miguel Fevereiro from Laboratório Nacional de Investigação Veterinária. LB Broth, used to culture *Escherichia coli (E. coli) *for plasmid production was also obtained from Sigma (Saint Louis, MO).

### Liposome and lipoplex preparation

Cationic liposomes were prepared at 0.5–4 mM concentration in cationic lipid as described elsewhere [29].

The plasmid was replicated in *E. coli *(DH5*α*) and purified employing a Qiagen Plasmid Midi Kit procedure (Valencia, CA). DNA concentration was measured spectrophotometrically and its purity and integrity were assessed using agarose gel electrophoresis. Working solutions of BOBO-1 were prepared immediately prior to use by diluting the DMSO stock solution with the working buffer. The dye/DNA solutions were prepared as described elsewhere [29].

The lipoplexes (cationic liposomes-DNA complexes) were obtained by direct and rapid addition of an appropriate amount of the cationic lipid dispersion to the DNA plasmid solution ([DNA] = 20 *μ*g/mL) at various charge ratios (DOTAP/DNA between 0.001 and 18), by adding rapidly equal volumes. The complexes were incubated at room temperature for 30 min, minimum, before use.

### Steady-state and lifetime fluorescence measurements

Fluorescence spectra and steady-state measurements were carried out with a SLM-Aminco 8100 Series 2 spectrofluorimeter (Rochester, NY; with double excitation and emission monochromators, MC-400) as described elsewhere [28].

Fluorescence decay measurements were carried out with a single photon-timing system described elsewhere [28]. Excitation of BOBO-1 was performed at 435 nm, using a Titanium-Saphire (Ti-Sph) laser. In these conditions, no protein interference was observed when serum solutions were analysed. Data analysis was carried out using non-linear, least squares iterative convolution method based on the Marquardt algorithm [30]. The goodness of the fits was judged from the chi-square values (*χ*^2^<1.3) and the lifetime-weighted quantum yields, <*τ*>, were calculated according to

$$<\tau >=\sum_{i=1}^{n}\alpha_{i}\times \tau_{i}$$

where *α*_*i *_and *τ*_*i *_are the normalized pre-exponential factor (amplitude) and the lifetime, respectively, in *n *exponential decay components.

### FRET assays

The lipoplexes' samples used in FRET assays were prepared with fluorescent dyes incorporated both in the liposomes and in the DNA. BOBO-1 was used as a donor and BODIPY-PC was the acceptor. Samples with a BOBO-1/DNA d/b ratio (dye molecules/base number) of 0.01 were prepared, allowing 1 h 30 min of incubation. Separately, several batches of liposomes were prepared with different BODIPY-PC concentrations with BODIPY-PC/DOTAP molar ratios ranging from 1:500 to 1:50. All the samples were protected from light since preparation until the end of measurements.

The decay of the donor in the presence of a plane of acceptors (*i*_DA_(*t*)), assuming low density of excited acceptors, no energy migration among donors, no translational diffusion of probes during the donor excited state lifetime, uniform distribution of acceptors, a single Förster distance *R*_0 _value for all donor-acceptor pairs, and probe dimensions <<*R*_0 _is faster than that in the absence of acceptors (*i*_D_(*t*) below), and is given by [29]:

$$i_{\text{DA}}(t)=(1-\gamma )i_{\text{D}}(t)\exp\left(-\frac{2C}{\Gamma (2/3)b}\int_{0}^{1}\frac{1-\exp(-tb^{3}\alpha^{6})}{\alpha^{3}}d\alpha \right)+\gamma i_{\text{D0}}(t)$$

**Figure 4:**
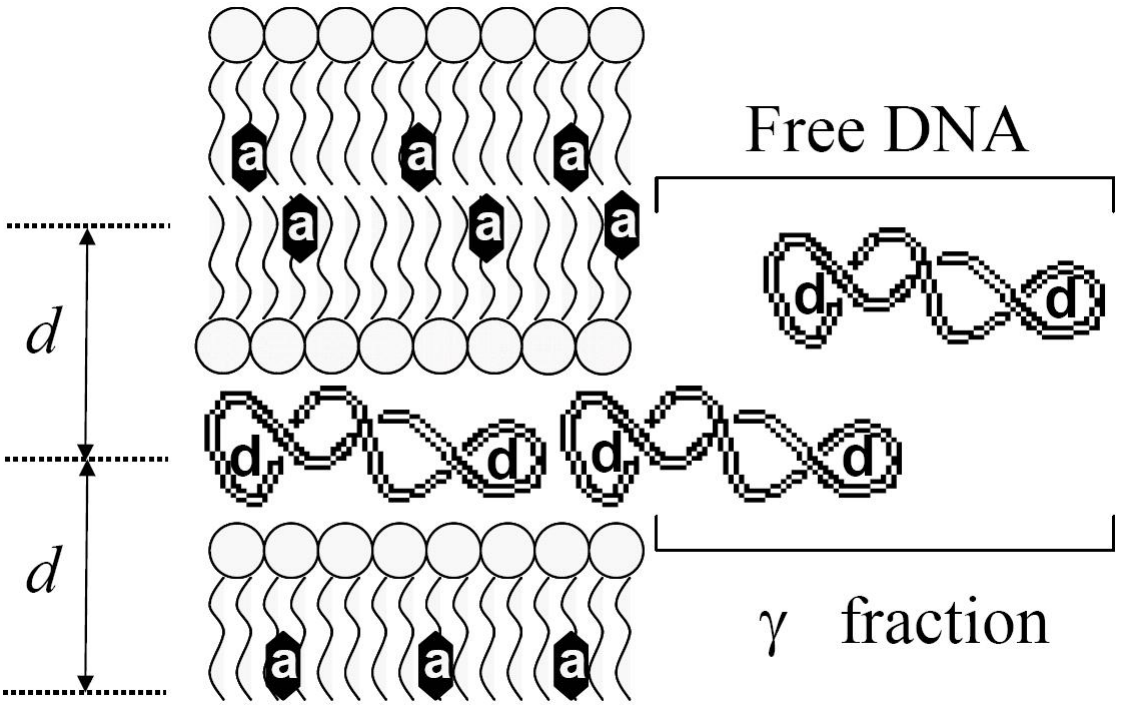
**Schematic representation of lipoplexes**. Lipoplex multilamellar structure with the acceptor probe (a) on the lipid (BODIPY-PC) and donor probe (d) on DNA (BOBO-1).

The *γ *value (see Fig. 4) is the fraction of donor molecules which decay (*i*_D0_(t)) is unaffected by the acceptors corresponding to the BOBO-1 molecules within free DNA. Additionally, *b *= (*R*_0_/*d*)^2^*τ*^-1/3^, where *d *is the distance between donor and acceptor planes, Γ is the complete Gamma function, and *C *is a variable proportional to the number of acceptor molecules per unit area in the acceptor plane (and is known beforehand [29]). The theoretical FRET efficiency is calculated by

$$E=1-\int_{0}^{\infty }i_{DA}\left(t\right)dt/\int_{0}^{\infty }i_{D}\left(t\right)dt$$

and can be compared with the experimental (both from steady-state and integrated time-resolved measurements) value, calculated using

$$E=1-\left(\frac{I_{DA}}{I_{D}}\right)$$

where *I*_DA _and *I*_D _are the donor fluorescence intensities in absence and in presence of acceptor, respectively. In this work, BOBO-1 quantum yields, obtained from the lifetime-weighted quantum yields [29] were used to calculate *R*_0 _values (given in Table 1). To this purpose, we only used BOBO-1 quantum yield values obtained for higher charge ratios, which become constant from a specific (+/-) value on, reflecting the environment of total coverage of the DNA by the lipid.

The methodology to assess the liposome complexation efficiency of a specific formulation (lipid/DNA) was as follows:

*(i) *A FRET assay with charge ratios ≥5, 10 or 14, depending on the system (charge ratios with absence of free DNA, and so *γ *= 0 was fixed) was analyzed to recover *d *= *R*_0_*b*^-1/2^*τ*^-1/6 ^as the sole parameter, using Eq. 2.

*(ii) *Fixing *d *to the value recovered in *(i)*, FRET assays of lipoplexes with lower charge ratios (with free DNA) enabled the recovery of the *γ *values. The liposome complexation efficiency (C.E.) was calculated for each case by:

C.E.= (1-*γ*) × 100

## Abbreviations

BOBO-1: benzothiazolium, 2,2'-[1,3- propanediylbis[(dimethyliminio)-3,1- propanediyl-1(4H)-pyridinyl-4- ylidenemethylidyne]]bis [3-methyl]-, tetraiodide; DOTAP: 1,2-dioleoyl-3-trimethylammonium-propane; FRET: Fluorescence Resonance Energy Transfer; PBS: Phosphate-buffer Saline; FBS: Fetal Bovine Serum.

## Authors' contributions

CM, LL, MP and MRA-B participated in the research design. CM and AF performed the research. MP, AF and MRA-B contributed with new reagents and analytic tools. CM and LL analyzed the data and CM and LL wrote the paper. All authors read and approved the final manuscript.
